# Supplementary material for: Senescent cancer-associated fibroblasts in pancreatic adenocarcinoma restrict CD8+ T cell activation and limit responsiveness to immunotherapy in mice
Source: Nat Commun. 2024 Jul 22;15:6162. doi: 10.1038/s41467-024-50441-7 (PMC11263607; doi:10.1038/s41467-024-50441-7)
Supplement: Supplementary file 1 — Supplementary Information [file 41467_2024_50441_MOESM1_ESM.pdf]

Supplementary Information

Senescent cancer-associated fibroblasts in pancreatic adenocarcinoma impair CD8+ T cell activation and responsiveness to immunotherapy in mice

Benjamin Assouline[1*], Rachel Kahn[1*], Lutfi Hodali[1*], Reba Condiotti[1], Yarden Engel[2], Ela Elyada[3], Tzlil Mordechai-Heyn[1,4], Jason R. Pitarresi[5,6], Dikla Atias[7], Eliana Steinberg[8], Tirza Bidany-Mizrahi[2], Esther Forkosh[9], Lior H. Katz[9], Ofra Benny[8], Talia Golan[7], Matan Hofree[2, 10], Sheila A. Stewart[11], Karine A. Atlan[12], Gideon Zamir[4], Ben Z. Stanger[13], Michael Berger[2] and Ittai Ben-Porath[1§]

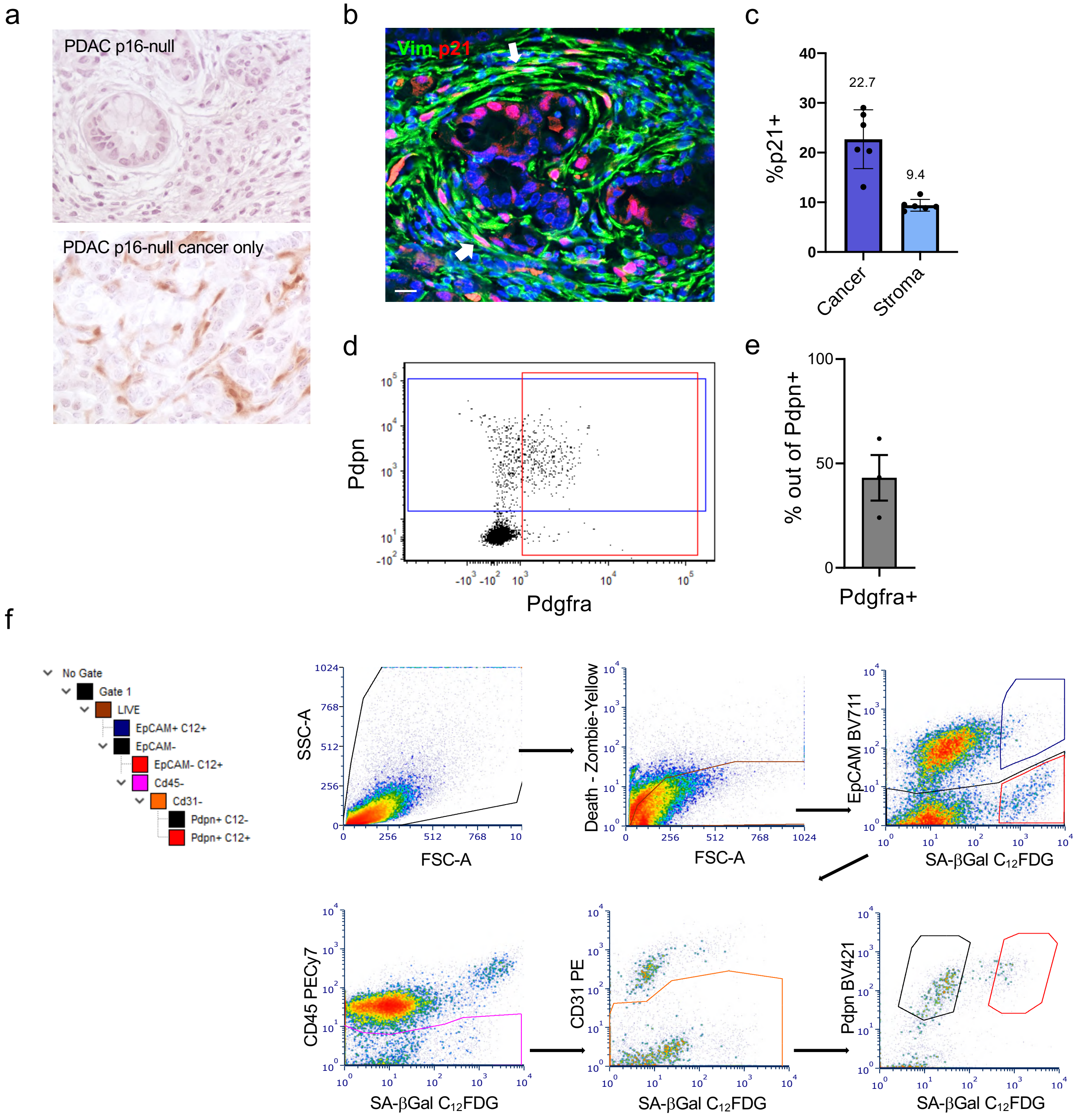

Supplementary Figure 1: Senescent CAFs in mouse pancreatic cancer lesions, additional data. **a)** Controls for p16 staining specificity. Pancreatic Kras-driven PDAC lesions from p16-null mice, or from mice carrying a conditional p16 null allele deleting p16 only in tumor cells, stained for p16. **b)** Co-stain of pancreatic Kras-driven lesions for p21 and the fibroblast marker vimentin (Vim). White arrow indicates p21+ CAFs. Scale bar=20µm. **c)** Percentage of p21+ cells in epithelial (cancer) and stromal compartments of pancreatic lesions in Kras mice. Mean of n=6 tumor regions from 2 mice ±SEM. **d)** FACS analysis of dissociated pancreas from mouse Kras-driven pancreas, stained for the CAF markers Pdpn and Pdgfra, showing that Pdgfra+ cells represent a subset of Pdpn+ CAFs. Chart shows Epcam- cells only. **e)** Percentage of Pdgfra+ cells out of Pdpn+ cells in pancreatic cells from Kras-driven lesions analyzed by FACS as in panel d. Mean of n=3 tumors ±SEM. **f)** Gating procedure for identification and isolation of mouse PDAC CAFs.

a

b

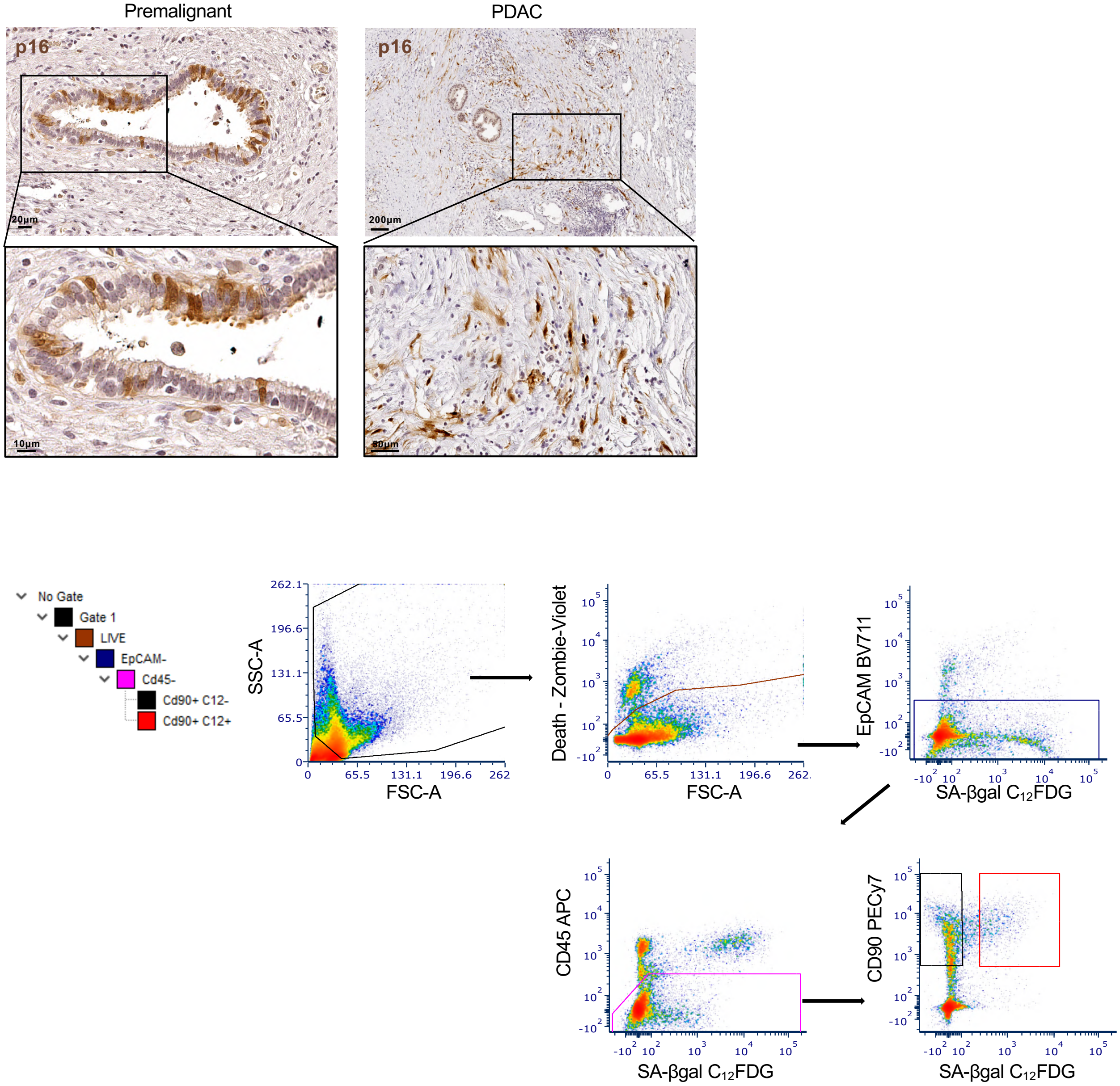

Supplementary Figure 2: Senescent CAFs in human pancreatic cancer lesions, additional data. **a)** Additional sections of human premalignant (PanIN, left) and PDAC (right) samples, stained for p16. PanIN tissue shows staining of epithelial cells. **b)** Gating procedure for identification and isolation of human PDAC CAFs.

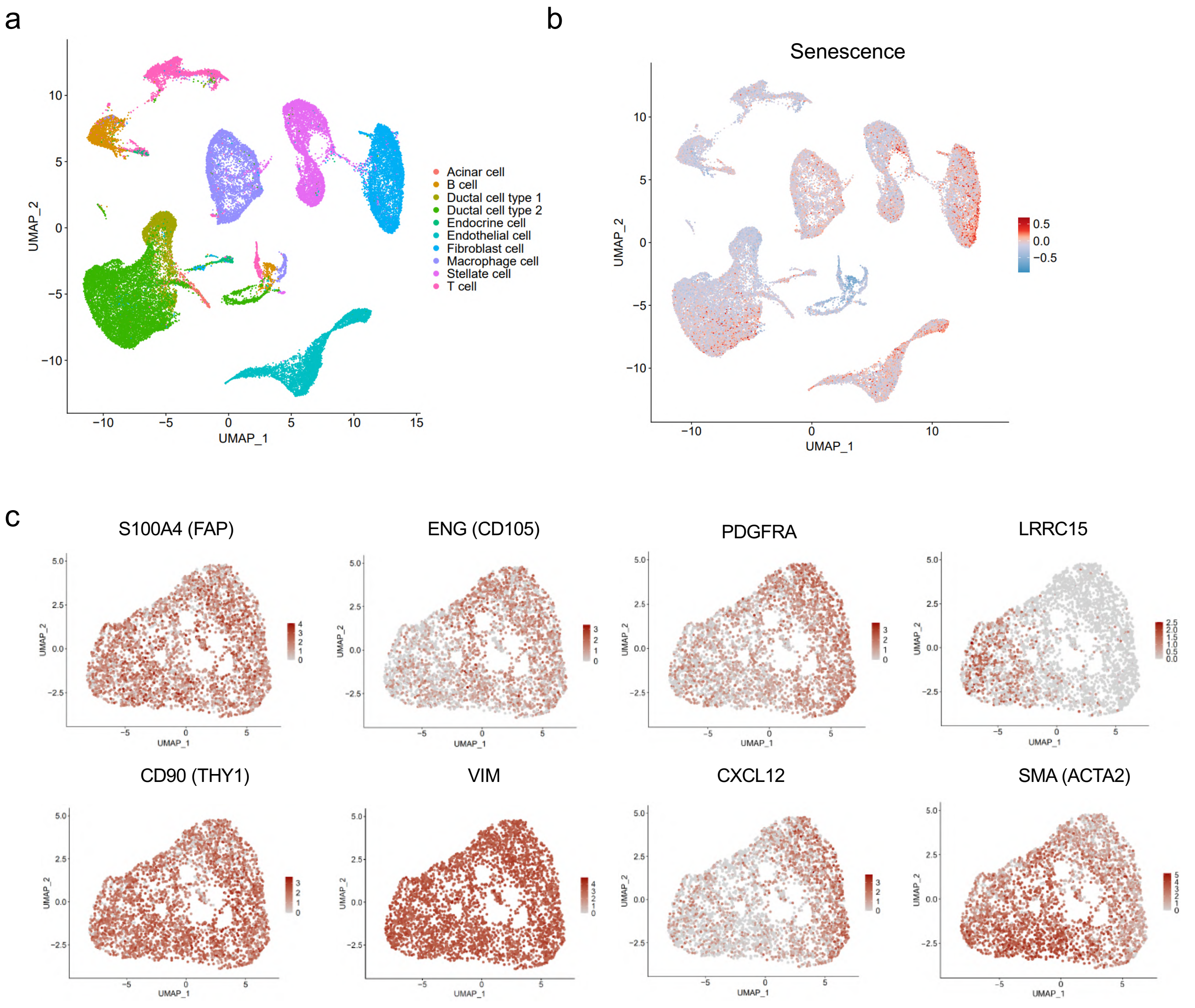

Supplementary Figure 3: Senescent cells in human PDAC scRNA-seq dataset. a) UMAP showing cell clusters representing different cell types identified within scRNA-seq dataset of human PDACs, obtained from Peng et al.. **b)** Relative expression score (red) of the senescence signature in different cell types in the same dataset. **c)** Relative expression (red) of different studied PDAC CAF markers within the cluster representing all CAFs.

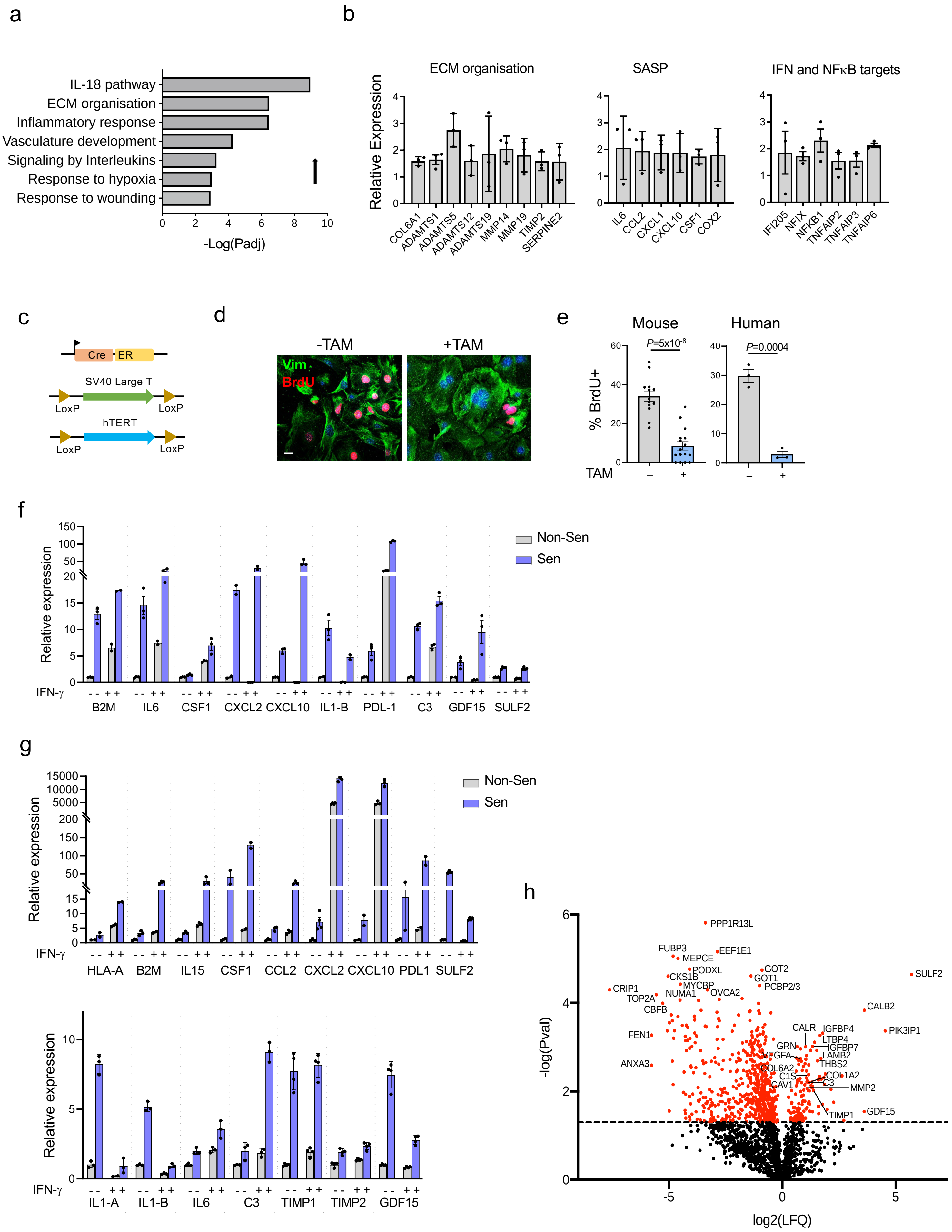
a
IL-18 pathway
ECM organisation
Inflammatory response
Vasculature development
Signaling by Interleukins
Response to hypoxia
Response to wounding
-Log(Padj)
b
ECM organisation
SASP
IFN and NFκB targets
Relative Expression
COL6A1
ADAMTS1
ADAMTS5
ADAMTS12
ADAMTS19
MMP14
MMP19
TIMP2
SERPINE2
IL6
CCL2
CXCL1
CXCL10
CSF1
COX2
IFI205
NFIX
NFKB1
TNFAIP2
TNFAIP3
TNFAIP6
c
Cre
ER
SV40 Large T
LoxP
LoxP
hTERT
LoxP
LoxP
d
-TAM
+TAM
Vim
BrdU
e
Mouse
Human
% BrdU+
P=5x10-8
P=0.0004
TAM
f
Relative expression
Non-Sen
Sen
IFN-γ
B2M
IL6
CSF1
CXCL2
CXCL10
IL1-B
PDL-1
C3
GDF15
SULF2
g
Relative expression
Non-Sen
Sen
IFN-γ
HLA-A
B2M
IL15
CSF1
CCL2
CXCL2
CXCL10
PDL1
SULF2
Relative expression
IFN-γ
IL1-A
IL1-B
IL6
C3
TIMP1
TIMP2
GDF15
h
-log(Pval)
log2(LFQ)
PPP1R13L
FUBP3
EEF1E1
MEPCE
PODXL
GOT2
CKS1B
GOT1
SULF2
CRIP1
MYCBP
PCBP2/3
NUMA1
OVCA2
TOP2A
CALB2
CBFB
PIK3IP1
CALR
IGFBP4
FEN1
LTBP4
GRN
IGFBP7
VEGFA
LAMB2
ANXA3
THBS2
COL6A2
C1S
COL1A2
CAV1
C3
MMP2
GDF15
TIMP1

Supplementary Figure 4: Senescent CAF gene expression profiles, additional data. a) Gene sets up-regulated in SA-βGal+ CAFs isolated from Kras+p53 mouse tumors, compared to matched SA-βGal- CAFs, as measured by mRNA-seq. x axis indicates $-\text{Log}_{10}$ *P*adj values, calculated by Metascape with Bonferroni method multiple hypothesis correction. **b)** Selected upregulated genes in the same SA-βGal+ CAFs, measured by mRNA-seq. y axis indicates fold change relative to SA-βGal- CAFs. Mean of n=3 tumors ±SEM, *P*adj<0.2, DESeq2, for all genes (Benjamini-Hochberg FDR correction). **c)** Vectors introduced into primary CAFs isolated from mouse and human PDACs to generate the inducible senescence system. Human CAFs were infected with two viruses, carrying the hTERT and SV40-LT genes, as well as with a virus carrying the CreER inducible recombinase. Mouse CAFs received the SV40-LT and CreER vectors. 4-OHT (TAM) treatment results in excision of the immortalizing gene and senescence activation. **d)** Mouse CAFs carrying the inducible senescence vectors, treated or untreated with TAM and stained for Vimentin (Vim) and the proliferation marker BrdU. **e)** Percentages of BrdU+ cells in CAFs carrying the inducible senescence vectors treated or untreated with TAM. Values indicate mean of n microscopic fields per sample, n=13,15 mouse, n=3,3 human, ±SEM, *t* test. **f)** Expression levels of genes encoding cytokines and other immune-modulatory genes in non-senescent (Non-Sen) and senescent (Sen) mouse PDAC CAFs, upon induction of senescence in culture, measured by qRT-PCR. Shown are levels in untreated cells, as well as in cells treated with IFNγ for 48 hours. Mean of n=2-3 replicates ±SEM. *t* test. All genes are significantly elevated in the senescent versus non-senescent cells, $P<0.05$, *t* test. **g)** Similar analysis of human PDAC CAFs, senescent and non-senescent. Mean of n=2-3 replicates ±SEM. All genes are significantly elevated in the senescent versus non-senescent cells, $P<0.05$, *t* test. **h)** Volcano plot representing protein content of conditioned media from human senescent CAFs versus non-senescent CAFs, analyzed by mass spectrometry. x axis represents $\log_2$ fold change of protein quantity (LFQ) in the senescent CAF CM, y axis represents $-\log_{10} P$ value of change. Selected proteins are indicated by name. n=4 samples in each group, collected in independent experiments.

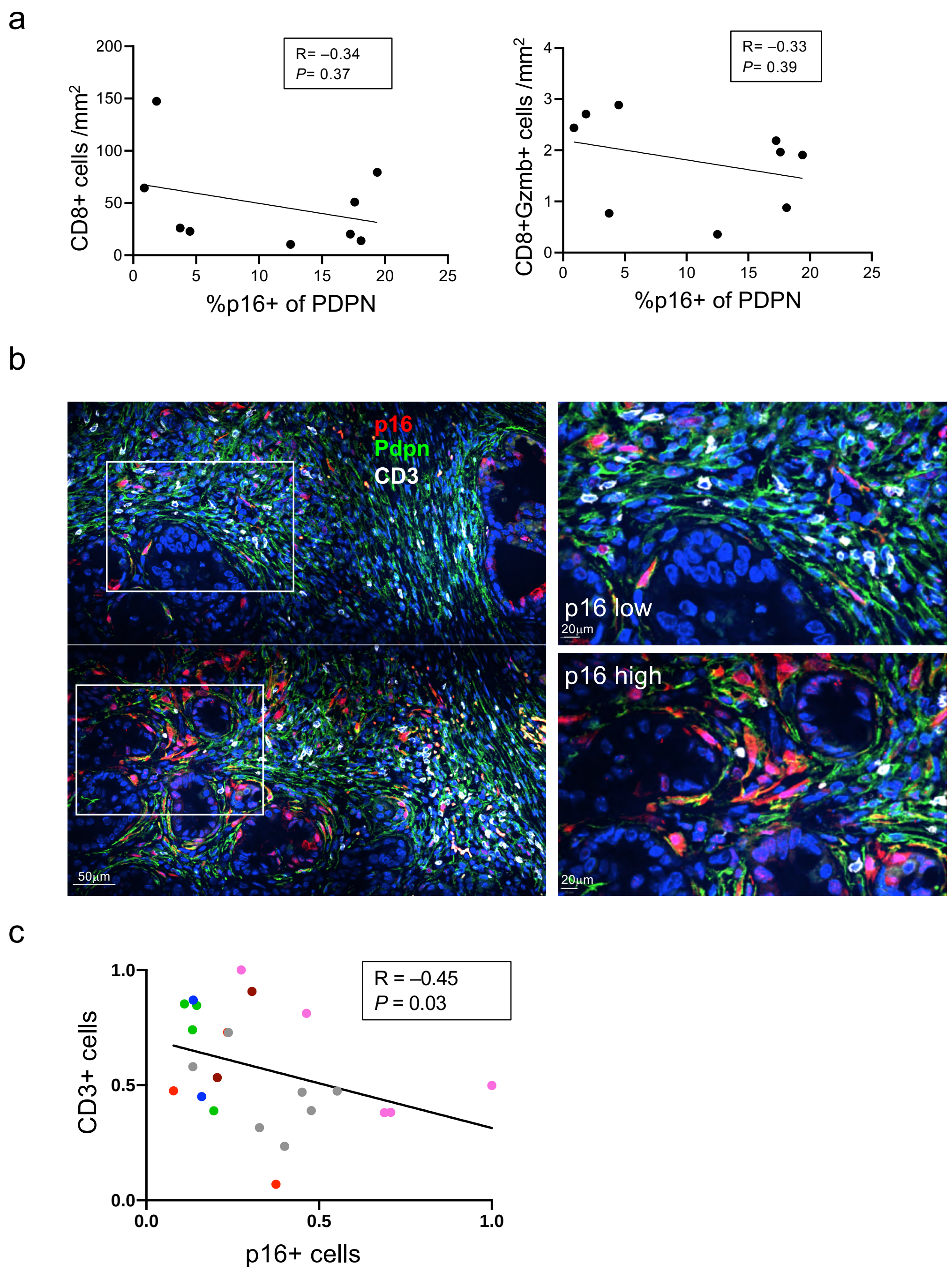

Supplementary Figure 5: Senescent CAF localization versus T cells, additional data. a) Correlations between percentage of p16+ cells out of CAFs, and CD8+ T cell numbers (left) or Gzmb+CD8+ cell numbers per mm^2, in individual tumors grown from distinct KPC lines, quantified by image analysis. R value indicate Pearson correlation with calculated *P* value, with line indicating linear regression. **b)** Section of representative pancreatic lesion in a Kras-expressing mouse, co-stained for p16, the CAF marker Pdpn, and CD3 marking T cells. Representative regions with high or low CAF p16 content and corresponding low and high T cell content are highlighted. **c)** Relative numbers of CD3+ cells versus p16+ stromal cells in Kras driven lesions as shown in panel b. Each dot represents different tumor region, with dot color indicating same tumor, scored by image analysis. Values represent image arbitrary units, normalized to highest values.

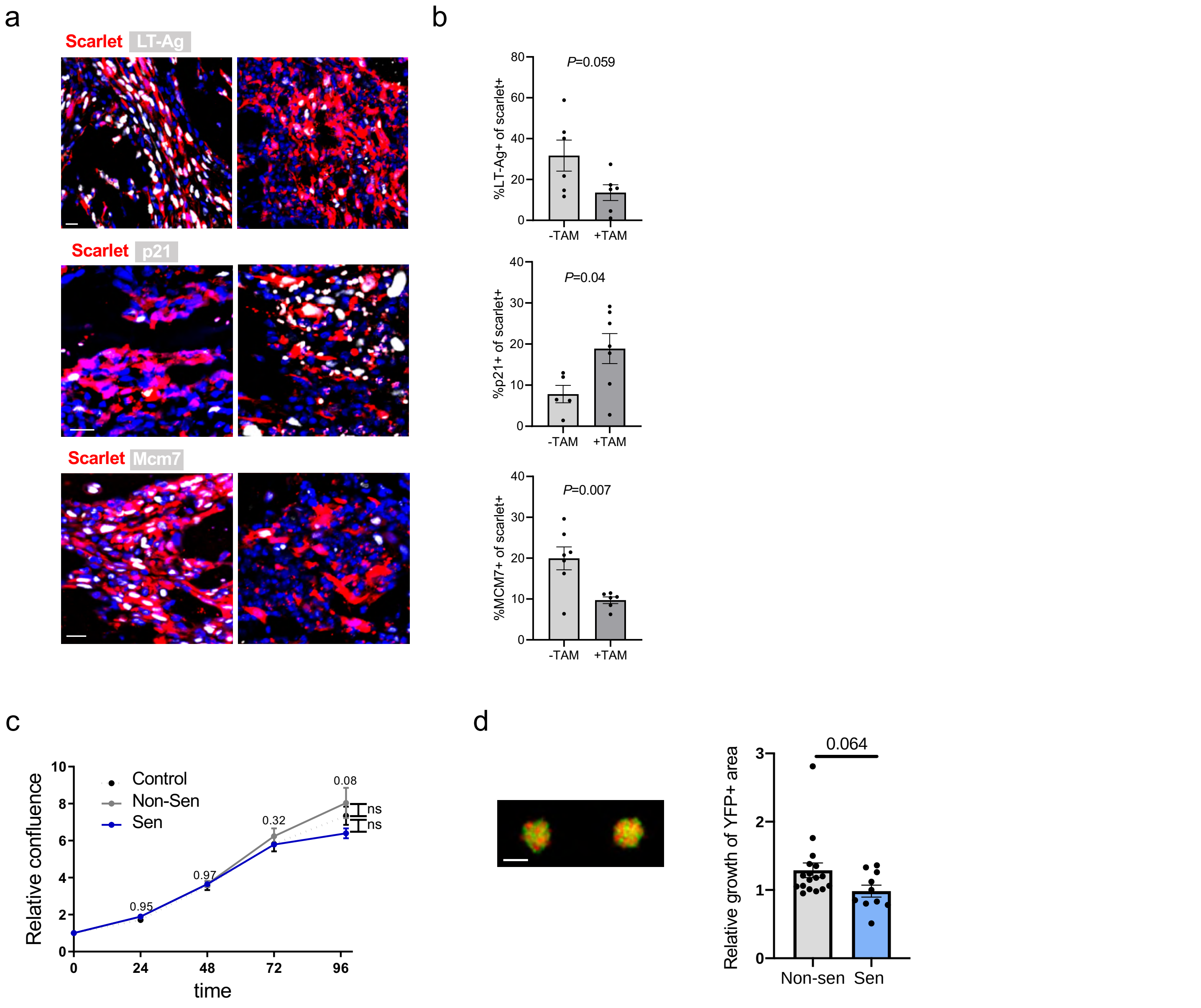

Supplementary Figure 6: Activation of CAF senescence in mice after transplantation, and effects of CAFs on PDAC cell proliferation. a) Lesion sections from untreated (-TAM) and tamoxifen-treated (+TAM) mice, one week after co-injection of CAFs and KPC tumor cells, stained for mScarlet (red) marking the injected CAFs, and for the indicated markers (white): large T-antigen (LT-Ag), p21 and the proliferation marker Mcm7. Scale bar=20µm. **b)** Percentages of mScarlet+ CAFs co-expressing the indicated markers in lesions as shown in panel a, one week after co-injection into the mice. Values indicate mean ±SEM, *t* test. n=6,6 tumors for large T-antigen stain, n=5,7 for p21 (one extreme outlier excluded from control group), n=7,6 for Mcm7 stain. **c)** Growth curves of X252 primary human PDAC cells cultured in the presence of conditioned media from non-senescent or senescent human PDAC CAFs. y axis indicates relative cell confluence measured by image analysis. n=6 replicates, numbers indicate *P* value, *t* test. **d)** Tumorspheres grown from a mix of YFP-labelled mouse KPC tumor cells (line 6555c3) and non-senescent and senescent mScarlet-labeled mouse CAFs, in a 1:4 ratio. Images show representative tumorspheres, graph shows relative sphere sizes, quantifying YFP+ area only. Mean of n=16,10 spheres ±SEM. *t* test. Scale bar=250µm.

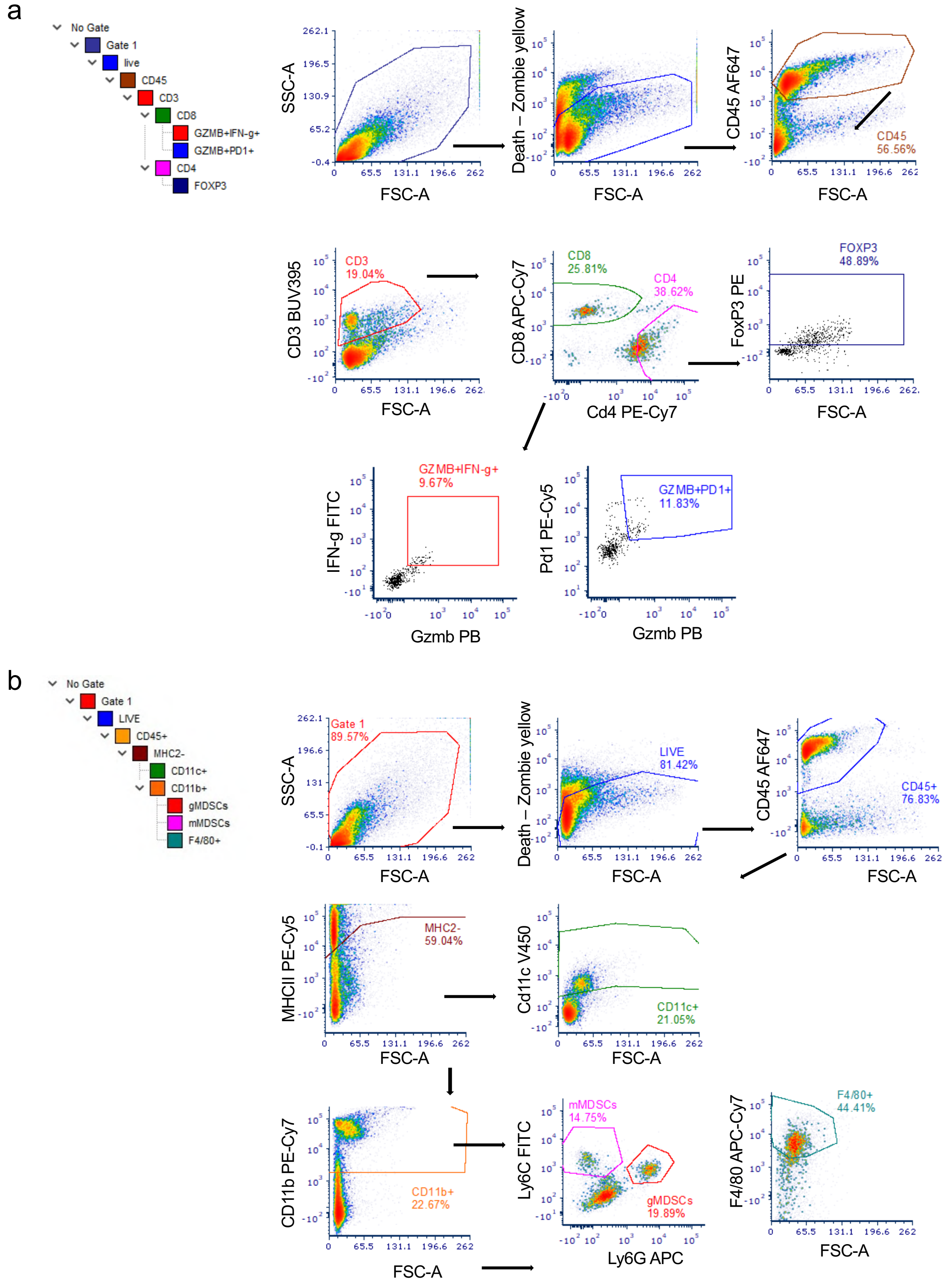

Supplementary Figure 7: FACS gating schemes for tumor immune cell analysis. a) Gating scheme for lymphocyte analysis of KPC tumors. **b)** Gating scheme for myeloid cell analysis of KPC tumors.

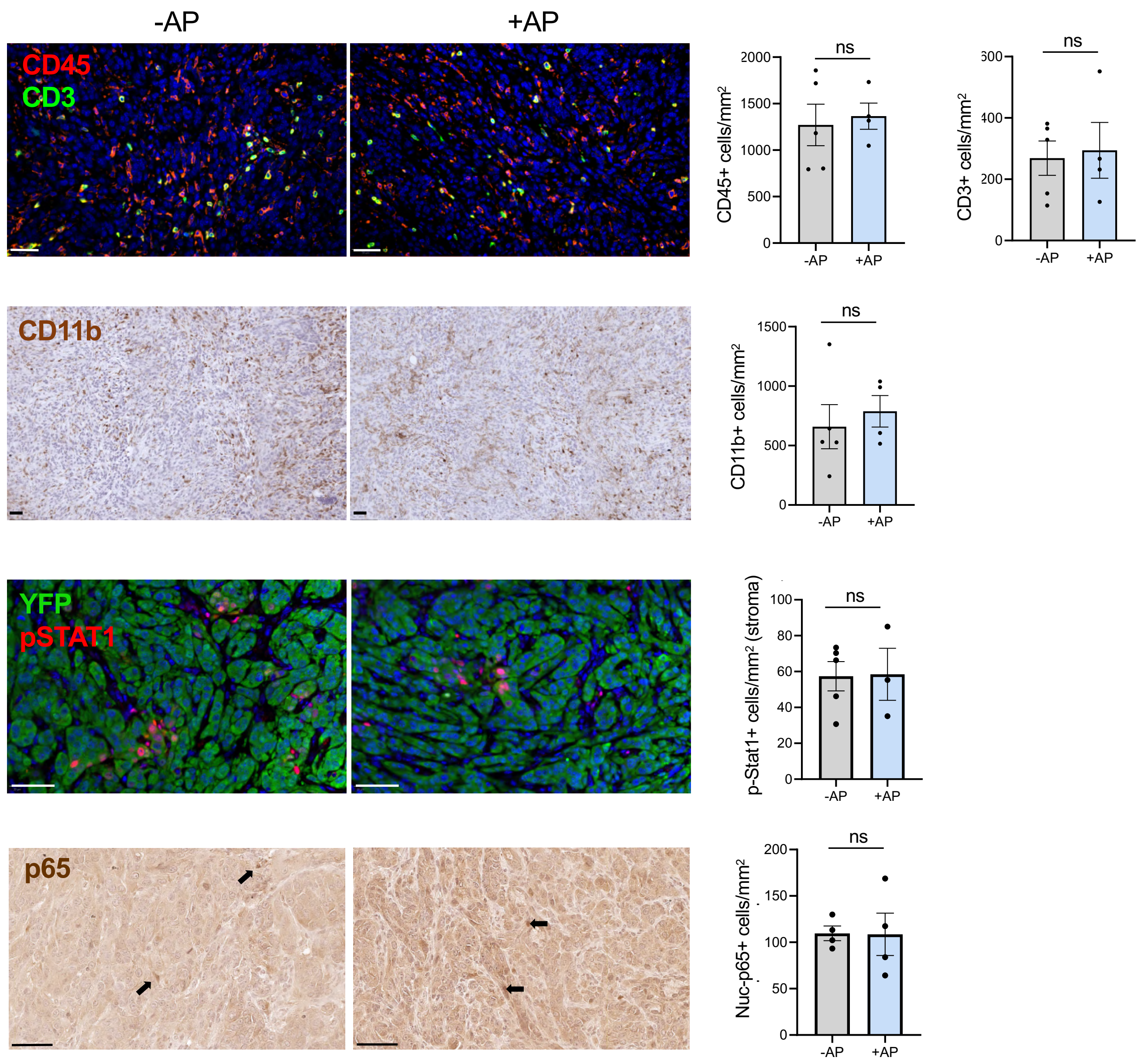

Supplementary Figure 8: Additional stains of KPC tumors in Ink-ATTAC model for immune and inflammation markers. Images on left show representative stains from untreated and AP-treated Ink-ATTAC mice implanted with 6422c1 KPC cells, analyzed in Figure 6. CD45 marks immune cells, CD3 marks T cells, CD11b marks myeloid cells, phospho-STAT1 and nuclear p65 (RelA) marks cells with activated inflammatory and interferon pathways. YFP marks the tumor cells. Scale bar=50µm. Graphs indicate numbers of scored cells in images, in control and treated mice, as indicated. Values indicate mean of n=5,4 tumors for CD45, CD3, CD11b, n=5,3 for p-STAT1, n=4,4 for p65 ±SEM**,** *t* test. ns – non-significant.

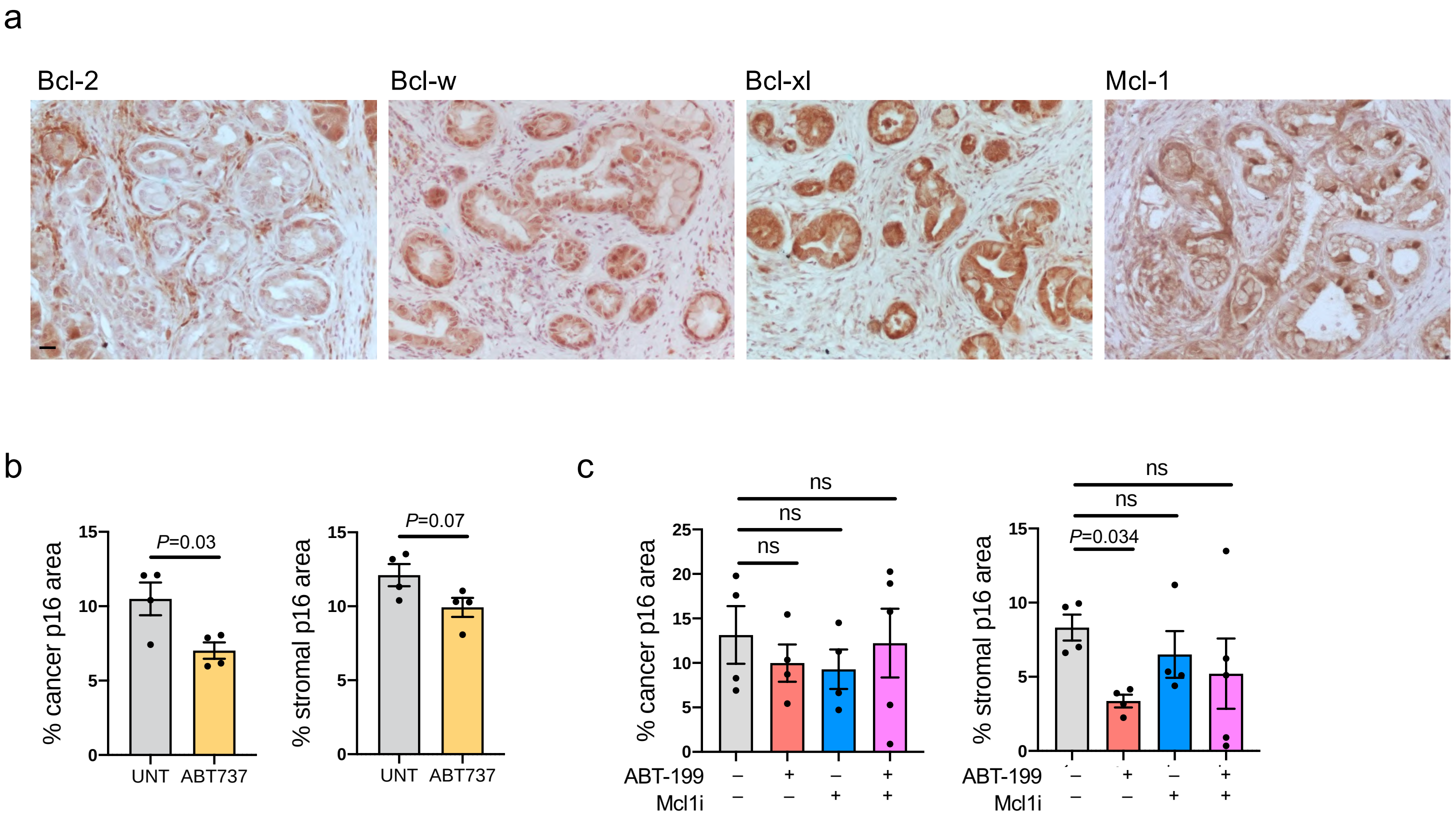

Supplementary Figure 9: Expression and senolytic targeting of different Bcl-2 family proteins in Kras-driven lesions. a) Sections of Kras-driven pancreatic lesions stained for the four indicated Bcl2 family proteins. Note that Bcl2 expression is most associated with stromal cells. Scale bar = 20µm. **b)** Quantification of p16+ cells in epithelial (cancer) and stromal regions of Kras-lesions grown in mice either untreated (UNT) or treated with ABT-737, which inhibits all Bcl2 family proteins except Mcl1. Mean of n=4 ±SEM, *t* test. **c)** Quantification of p16+ cells in epithelial (cancer) and stromal regions of Kras-lesions grown in mice either untreated (UNT), treated with the Bcl2 inhibitor ABT-199, the Mcl1 inhibitor S63845 (Mcl1i), or with both. Note that ABT-199 was most effective in eliminating stromal p16+ cells. Mice were treated 3 times over a week, after 10 months of KRAS induction. Mean of n=4,4,4,5 ±SEM. Values indicate mean ±SEM. *P* values were calculated with Brown-Forsythe ANOVA test and Benjamini-Hochberg false discovery rate correction. ns – non significant.

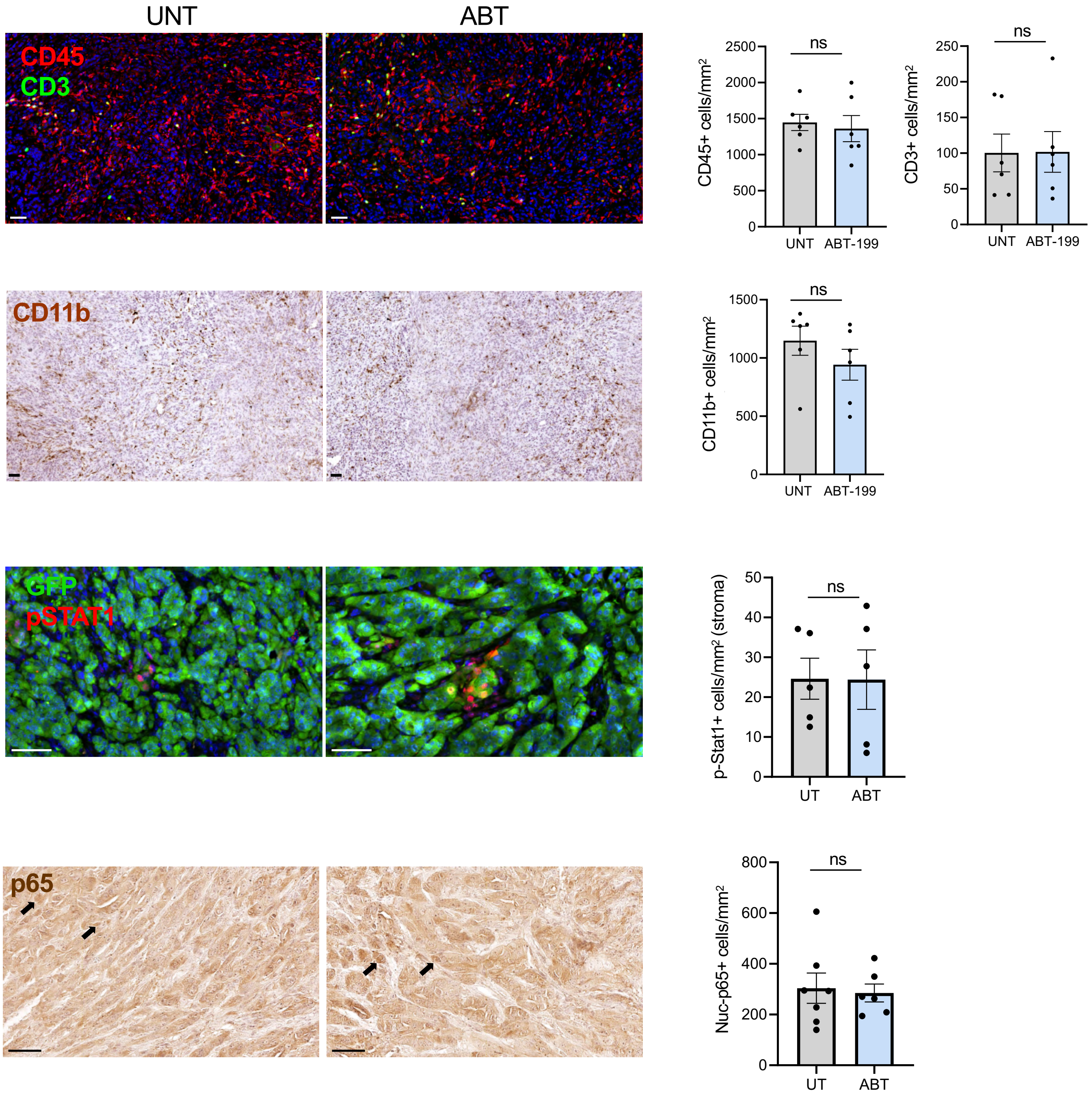

Supplementary Figure 10: Additional stains of KPC tumors treated with ABT-199 for immune and inflammation markers. Images on left show representative stains from untreated and ABT-199-treated mice implanted with 6422c1 KPC cells, analyzed in Figure 7. CD45 marks immune cells, CD3 marks T cells, CD11b marks myeloid cells, phospho-STAT1 and nuclear p65 (RelA) marks cells with activated inflammatory and interferon pathways. YFP marks the tumor cells. Scale bar=50µm. Graphs indicate numbers of scored cells in images, in control and treated mice, as indicated. n=6 tumors for CD45, CD3, CD11b; n=5 for p-Stat1, n=7,6 for p65. Values indicate mean ±SEM**,** *t* test. ns – non-significant.

Supplementary Table 1. Human patient sample details

a) Human premalignant and PDAC samples used for immunohistochemistry

				p16 stain levels and location:	
	Sex	Age	Pathology	p16 in cancer 0-3	p16 in stroma 0-3
Premalignant-Midgam	female	75	Intraductal papillary mucinous neoplasm (IPMN) with low grade dysplasia	1	0
	male	75	IPMN with low grade dysplasia	1	1
	female	49	IPMN with low grade displasia, PanINs and inflammation	1	0
	male	57	IPMN	3	0
	female	78	PDAC moderately to poorly differentiated and IPMN	3	1
	female	69	Neuroendocrine tumor and IPMN with low grade displasia	2	0
	female	69	Benign Neuroindocrine tumor (Islet cell tumor) and IPMN with low grade displasia	2	2
	female	73	Pancreas shows fibrosis, atrophy of exocrine part, chronic inflammation and foci of IPMN with low grade dysplasia.	3	2
	female	68	Well differentiated PDAC and IPMN with high grade dysplasia	3	2
	male	63	IPMN with low grade dysplasia and serous cystadenoma	3	1
	female	65	IPMN with low grade dysplasia	3	0
	male	35	IPMN with low-grade dysplasia	1	0
Premalignant	female	48	IPMN with low grade dysplasia, branch-duct type, gastric epithelium	2	2
	male	69	Pancreatic adenocarcinoma poorly differentiated arising in IPMN, gastric type	3	3
	female	48	IPMN, gastric type	2	2
	male	65	IPMN with low to intermediate dysplasia, intestinal type	3	3
	male	58	Cystic lesion, cystic intraductal mucinous neoplasm with low grade dysplasia and PanIN-1b	2	2
				p16 stain levels and location:	
	Sex	Age	Pathology	p16 in cancer 0-3	p16 in stroma 0-3
PDAC	male	81	PDAC, moderately to poorly differentiated	2	2
	female	68	Adenosquamous carcinoma of pancreas, moderately differentiated	0	2
	male	64	PDAC, well differentiated	1	3
	female	82	PDAC, moderately to poorly differentiated	0	2
	female	77	Adenosquamous carcinoma of pancreas, poorly differentiated	1	1
	male	58	PDAC, moderately to poorly differentiated	2	1
	female	65	Adenosquamous carcinoma of pancreas, moderately differentiated	1	2
	male	68	PDAC, moderately to poorly differentiated	1	2
	male	43	PDAC, poorly differentiated	0	3
	female	69	PDAC, well differentiated	1	0
	male	79	PDAC, moderately differentiated	2	0
	male	69	PDAC, poorly differentiated	3	2

b) Human PDAC samples from pancreatectomy surgeries

Used for CAF isolation by FACS:

Surgery Date	Diagnosis	Tumor location	Surgery type	Prior treatment	Sex	Age
30.12.21	PDAC	head	Pancreaticoduodenectomy	Neoadjuvant: chemo and radiation	unknown	unknown
20.01.22	PDAC	tail	Distal pancreatectomy	Neoadjuvant: chemo	unknown	unknown
20.02.22	PDAC	tail	Distal pancreatectomy	Patient after whipple, cancer recurrence, 2nd surgery after radiation	Male	76
07.07.22	PDAC	head	Pancreaticoduodenectomy		Female	83
21.07.22	PDAC	tail	Distal pancreatectomy	After radiation	Male	82

Used for establishment of cultured CAFs and tumor cell line:

Surgery Date	Diagnosis	Tumor location	Surgery type	Prior treatment	Sex	Age	
15/03/2020	PDAC	head	pancreas surgery	naïve	female	unknown	Tumor-derived CAFs
07/06/2016	PDAC	head	pancreas CNB	gemcitabine/GEMZAR	male	unknown	PDX-derived culture

Supplementary Table 2: Gene signatures used in scRNA-seq analysis

a) CAF subtype signatures:

myCAFs	iCAFs	apCAFs
TAGLN	IL6	SLPI
ACTA2	PDGFRA	CALCA
MMP11	CFD	MSLN
PDGFRB	PLA2G2A	SAA3P
HOPX	HAS1	CLU
POSTN	CXCL2	HLA-DRA
MYH11	CCL2	CD74
	CLU	CCL2
	EMP1	APOE
	LMNA	KRT19
	KLF4	
	CXCL12	
	C3	
	PTGDS	

b) For Senescence score:

Senescence Markers	Cell Cycle Genes		
CDKN2A	ORC3	ABL1	ORC6
CDKN2B	EP300	E2F1	CCND1
CDKN1A	ANAPC4	CDK7	ATM
CDKN1B	ANAPC11	CHEK2	ORC4
SERPINE1	STAG2	MCM2	CDK4
	ANAPC2	MCM4	MCM5
	ORC2	RB1	ANAPC1
	MDM2	DBF4	SFN
	PLK1	ANAPC5	RBL2
	SKP1	CDK2	E2F5
	MCM7	E2F2	MYC
	STAG1	MCM3	ORC5
	YWHAQ	ANAPC7	E2F3
	CHEK1	CDK6	GSK3B
	SKP2	PCNA	ANAPC10
	SMC1B	MCM6	RBL1
	ORC1	HDAC1	FZR1
	ATR	RAD21	

Supplementary Table 3. Antibodies and primers used in the study.

a) Antibodies used for immunohistology:

Mouse:

	**company**	**cat#**	**conc.**
p16	Abcam	211542	1/100
p16	Abcam	252788	1/100
CD3	BIORAD	MCA1477	1/100
CD8a	Invitrogen	14-0808-82	1/200
GZMB	Abcam	ab4059	1/300
Ki67	Abcam	ab16667	1/200
Pdpn	R&D systems	AF3244	1/200
CK8	PROGEN	GP-K8	1/100
Vimentin	PROGEN	GP53	1/100
PDGFRA	R&D systems	AF1062	1/25
GFP	Abcam	ab6673	1/400
DsRed (scarlet)	Takara	632496	1/500
Bcl2	BD Biosciences	610539	1/100
α-SMA	Sigma	ab2547	1/500
BrdU	BIORAD	1702	1/200
CK19	Abcam	ab52625	1/200
BCL-W	Cell Signalling	2724S	1/500
BCL-XL	Cell Signalling	2764S	1/1000
CD11b	Abcam	ab133357	1/4000
Foxp3	Thermo Fisher Scientific	14-5773-82	1/100
Mcm7	Santa Cruz	sc-56324	1/100
Large T-Ag	BD-Pharmingen	554149	1/100
Mcl-1	Proteintech	16225-1-AP	1/3000
CD45	Abcam	ab10558	1/150
CD45	BD-Pharmingen	550539	1/50
Mac-2	Biolegend	125401	1/50
cd31	R&D systems	AF3628	1/100
Phospho-Stat1	Cell Signaling	9167S	1/100
RELA/NFκB p65	Santa Cruz	sc-8008	1/500

Human:

	**company**	**cat#**	**conc.**
p16	Bio SB	BSB 3479	1/50
CK18	Thermo	MS-142-P	1/100
Vimentin	Progen	GP53	1/100
CD8a	Invitrogen	14-0085-80	1/100

b) Antibodies used for FACS:

Mouse:

	company	cat#	conc.
Zombie Yellow	BioLegend	77168	1/100
CD45 AF647	BioLegend	103123	1/500
CD45 PE	BioLegend	103105	1/500
CD45 PECy5	BioLegend	103109	1/500
CD3e BUV395	BD Biosciences	563565	1/100
CD8a APC/Cy7	BioLegend	100713	1/200
CD11b PE/Cy7	BioLegend	101215	1/500
CD11c PB	BioLegend	117321	1/100
MHC class II PE/Cy5	BioLegend	107611	1/500
Foxp3 PE	BD Pharmingen	560414	1/200
Granzyme B PB	BioLegend	515407	1/100
IFN-γ FITC	BioLegend	505805	1/200
IFN-γ Pe/Cy7	BioLegend	505825	1/200
Ly-6C FITC	BioLegend	128005	1/100
Ly-6G APC	BioLegend	127613	1/100
F4/80 APC/Cy7	BioLegend	123117	1/100
PD1 PE/Cy5	BioLegend	135255	1/200
CD4 PE/Cy7	BioLegend	100527	1/200
CD4 FITC	BioLegend	100527	1/200
EpCAM BV711	BioLegend	118233	1/100
CD31 PE	BioLegend	102508	1/200
Pdpn BV421	BioLegend	127423	1/200
PDGFRa (CD140a) PE/Cy5	BioLegend	135919	1/200

Human:

	company	cat#	conc.
IFN-γ PE	BD Biosciences	561056	1/100
CD8 BV711	BioLegend	344733	1/500
CD25 APC	BioLegend	302609	1/500
CD4 APC/Cy7	BioLegend	317417	1/500
Granzyme B PB	BioLegend	515407	1/100
CD90 PE/Cy7	BioLegend	328123	1/20
CD45 APC	BioLegend	304011	1/20
EpCam BV711	BioLegend	324239	1/20
PDPN PE	BioLegend	337003	1/20

c) Primers used for qRT-PCR:

**mouse**	**forward**	**reverse**
GAPDH	TCTTGTGCAGTGCCAGCCT	CCAATACGG CCAAATCCGT
B-ACTIN	CACAGCTTCTTTGCAGCTCCT	GTCATCCATGGCGAACTGG
B2M	GTATGCTATCCAGAAAACCC	CTGAAGGACATATCTGACATC
IL6	GTCTATACCACTTCACAAGTC	TGCATCATCGTTGTTCATAC
CSF1	TAGAAAGGATTCTATGCTGGG	CTCTTTGGTTGAGAGTCTAAG
CXCL2	GCTGTCAATGCCTGAAGA	CAGAAGTCATAGCCACTCTCA
CXCL10	AAGTGCTGCCGTCATTTTCT	TATGGCCCTCATTCTCACTG
PDL-1	TACAAGCGAATCACGCTGAA	AGCTTCTGGATAACCCTCGG
C3	TCCAACAAGAACACCCTCA	GGCTGGATAAGTCCCACA
IL1-B	ACCTTCCAGGATGAGGACATGA	CTAATGGGAACGTCACACACCA
GDF15	CACTGCAGACTTATGATGAC	AAATACACAATCCATCCACC
SULF2	TGAACAATACAGGCAGTTTC	TTCCAACATTCATCTTCTGG
p16 (for Taqman)	CGGTCGTACCCCGATTCAG	GCACCGTAGTTGAGCAGAAGAG
Probe p16 TaqMan	AAC GTT GCC CAT CAT CA	

**human**	**forward**	**reverse**
GAPDH	TCACCACCATGGAGAAGC	GCTAAGCAGTTGGTGGTG
B-ACTIN	GAGCACAGAGCCTCGCCTTT	TCATCATCCATGGTGAGCTGG
HLA-A	GAAGAGCTCCAGATAGAAAAGG	CTTTGCAGAAACAAAGTCAG
B2M	TCTCTCTTTCTGGCCTGGAG	AATGTCGGATGGATGAAACC
IL6	GGCATCTCAGCCCTGAGAAAG	CCAGGCAAGTCTCCTCATTGA
IL15	AGCAATGTTCCATCATGTTC	ATACGATCTTGTATGGGCTG
CSF1	TTAAGAAGGCATTTCTCCTG	CCTTGTCATGCTCTTCATAATC
CCL2	CTCAAACTGAAGCTCGCA	GTGACTGGGGCATTGATT
CXCL2	CATCGAAAAGATGCTGAAAAATG	CTTCAGGAACAGCCACCAATA
CXCL10	AGGAGTACCTCTCTCTAGAAC	AAAGACCTTGGATTAACAGG
PDL-1	ATGCCCCATACAACAAAATC	GACATGTCAGTTCATGTTCAG
C3	GCTGAAGGAAAAGGCCAAG	CGGTGCTGGTTTTATGGTG
IL1-A	AGAGGAAGAAATCATCAAGC	TTATACTTTGATTGAGGGCG
IL1-B	CTAAACAGATGAAGTGCTCC	GGTCATTCTCCTGGAAGG
TIMP1	CACCTTATACCAGCGTTATG	TTTCCAGCAATGAGAAACTC
TIMP2	GGCCTGAGAAGGATATAGAG	CTTTCCTGCAATGAGATATTCC
GDF15	CGAAGACTCCAGATTCCG	ACTTCTGGCGTGAGTATC
SULF2	CTTAAAGATGGAGGAAGCTATG	CAGTGATTTGGAAGAAGGTC
p16	CCCAACGCACCGAATAGTTA	ACCAGCGTGTCCAGGAAG
