# Supplementary material for: Senescent cancer-associated fibroblasts in pancreatic adenocarcinoma restrict CD8+ T cell activation and limit responsiveness to immunotherapy in mice
Source: Nat Commun. 2024 Jul 22;15:6162. doi: 10.1038/s41467-024-50441-7 (PMC11263607; doi:10.1038/s41467-024-50441-7)
Supplement: Supplementary file 3 — Reporting Summary [file 41467_2024_50441_MOESM3_ESM.pdf]

nature portfolio

Corresponding author(s): Ittai Ben-Porath

Last updated by author(s): 2023-8-10

Reporting Summary

Nature Portfolio wishes to improve the reproducibility of the work that we publish. This form provides structure for consistency and transparency in reporting. For further information on Nature Portfolio policies, see our Editorial Policies and the Editorial Policy Checklist.

Statistics

For all statistical analyses, confirm that the following items are present in the figure legend, table legend, main text, or Methods section.

n/a	Confirmed	
☐	☒	The exact sample size (*n*) for each experimental group/condition, given as a discrete number and unit of measurement
☐	☒	A statement on whether measurements were taken from distinct samples or whether the same sample was measured repeatedly
☐	☒	The statistical test(s) used AND whether they are one- or two-sided<br>*Only common tests should be described solely by name; describe more complex techniques in the Methods section.*
☒	☐	A description of all covariates tested
☐	☒	A description of any assumptions or corrections, such as tests of normality and adjustment for multiple comparisons
☐	☒	A full description of the statistical parameters including central tendency (e.g. means) or other basic estimates (e.g. regression coefficient) AND variation (e.g. standard deviation) or associated estimates of uncertainty (e.g. confidence intervals)
☐	☒	For null hypothesis testing, the test statistic (e.g. *F*, *t*, *r*) with confidence intervals, effect sizes, degrees of freedom and *P* value noted<br>*Give P values as exact values whenever suitable.*
☒	☐	For Bayesian analysis, information on the choice of priors and Markov chain Monte Carlo settings
☒	☐	For hierarchical and complex designs, identification of the appropriate level for tests and full reporting of outcomes
☐	☒	Estimates of effect sizes (e.g. Cohen's *d*, Pearson's *r*), indicating how they were calculated

Our web collection on statistics for biologists contains articles on many of the points above.

Software and code

Policy information about availability of computer code

Data collection	Microscopy - NIS Elements<br>FACS - BD FACSDiva.<br>Mass spectrometry - Exploris 480 mass spectrometer (Thermo)
Data analysis	Microscopy - NIS Elements (Nikon) and QuPath<br>FACS - FCS Express 7<br>mRNA-Seq - DESeq2<br>scRNA-seq - Seurat v4<br>Gene set enrichment analyses - Metascape, GSEA<br>Mass spectrometry - MaxQuant 2.1.1.0

For manuscripts utilizing custom algorithms or software that are central to the research but not yet described in published literature, software must be made available to editors and reviewers. We strongly encourage code deposition in a community repository (e.g. GitHub). See the Nature Portfolio guidelines for submitting code & software for further information.

Data

Policy information about availability of data

All manuscripts must include a data availability statement. This statement should provide the following information, where applicable:

- Accession codes, unique identifiers, or web links for publicly available datasets
- A description of any restrictions on data availability
- For clinical datasets or third party data, please ensure that the statement adheres to our policy

The mRNA-seq expression profiles generated in this study have been deposited in the Gene Expression Omnibus (GEO) database under accession number GSE235246, https://www.ncbi.nlm.nih.gov/geo/query/acc.cgi?acc=GSE235246. The mass spectrometry data have been deposited in the Open Science Framework (OSF) database, under DOI 10.17605/OSF.IO/28BZM, https://osf.io/28bzm/. The scRNA-Seq data generated by others and analyzed in this study is available at the Genome Sequence Archive (GSA), accession number CRA001160, project: PRJCA001063, https://ngdc.cncb.ac.cn/gsa/browse/CRA001160. Source data are provided with this paper.

Research involving human participants, their data, or biological material

Policy information about studies with human participants or human data. See also policy information about sex, gender (identity/presentation), and sexual orientation and race, ethnicity and racism.

Reporting on sex and gender

Pancreatic cancer samples were collected based on availability, without taking patient sex into account. Patients sex is reported for samples tested.

Reporting on race, ethnicity, or other socially relevant groupings

These parameters were not taken into account and reported. Samples are a random representation of pancreatic cancer patient population in Israel.

Population characteristics

Available age and clinical information are reported in the manuscript and listed below.

Sex Age Pathology
female 75 Intraductal papillary mucinous neoplasm (IPMN) with low grade dysplasia
male 75 IPMN with low grade dysplasia
female 49 IPMN with low grade displasia, PanINs and inflammation
male 57 IPMN
female 78 PDAC moderately to poorly differentiated and IPMN
female 69 Neuroendocrine tumor and IPMN with low grade displasia
female 69 Benign Neuroindocrine tumor (Islet cell tumor) and IPMN with low grade displasia
female 73 Pancreas shows fibrosis, atrophy of exocrine part, chronic inflammation and foci of IPMN with low grade dysplasia.
female 68 Well differentiated PDAC and IPMN with high grade dysplasia
male 63 IPMN with low grade dysplasia and serous cystadenoma
female 65 IPMN with low grade dysplasia
male 35 IPMN with low-grade dysplasia
female 48 IPMN with low grade dysplasia, branch-duct type, gastric epithelium
male 69 Pancreatic adenocarcinoma poorly differentiated arising in IPMN, gastric type
female 48 IPMN, gastric type
male 65 IPMN with low to intermediate dysplasia, intestinal type
male 58 Cystic lesion, cystic intraductal mucinous neoplasm with low grade dysplasia and PanIN-1b

Sex Age Pathology
male 81 PDAC, moderately to poorly differentiated
female 68 Adenosquamous carcinoma of pancreas, moderately differentiated
male 64 PDAC, well differentiated
female 82 PDAC, moderately to poorly differentiated
female 77 Adenosquamous carcinoma of pancreas, poorly differentiated
male 58 PDAC, moderately to poorly differentiated
female 65 Adenosquamous carcinoma of pancreas, moderately differentiated
male 68 PDAC, moderately to poorly differentiated
male 43 PDAC, poorly differentiated
female 69 PDAC, well differentiated
male 79 PDAC, moderately differentiated
male 69 PDAC, poorly differentiated

Recruitment

Samples were collected based on sample availability

Ethics oversight

Patient samples were obtained from the Hadassah-Hebrew University Medical Center, Jerusalem, Israel, Sheba Medical Center Tel-Hashomer, Israel, and from the Midgam, the Israeli Biorepository Network for Research. Peripheral blood samples were drawn from healthy adult volunteers at Hadassah Medical Center. All experiments were conducted under approval of the institutional Helsinki Committees, with written informed consent, numbers HMO-136-22, HMO-19-0024 (Hadassah) and 5073-18-SMC, 5539-08-SMC (Sheba).

Note that full information on the approval of the study protocol must also be provided in the manuscript.

Field-specific reporting

Please select the one below that is the best fit for your research. If you are not sure, read the appropriate sections before making your selection.

☒ Life sciences ☐ Behavioural & social sciences ☐ Ecological, evolutionary & environmental sciences

For a reference copy of the document with all sections, see nature.com/documents/nr-reporting-summary-flat.pdf

Life sciences study design

All studies must disclose on these points even when the disclosure is negative.

Sample size	Sample sizes were determined based on mouse cohort availability for transgenic lines and on experimental feasibility in xenograft transplantaion and mouse treatment experiments. Experiments were repeated to increase sample sizes. Technical replicates were determined based on standard experimental practices for the methods used.
Data exclusions	Replicates were excluded only in cases in which technical failure was evident.
Replication	All results shown were successfully replicated at least twice.
Randomization	Mice were allocated to groups randomly, maintaining similar sex and age distribution between experimental groups.
Blinding	No blinded allocation was conducted.

Reporting for specific materials, systems and methods

We require information from authors about some types of materials, experimental systems and methods used in many studies. Here, indicate whether each material, system or method listed is relevant to your study. If you are not sure if a list item applies to your research, read the appropriate section before selecting a response.

Materials & experimental systems

n/a	Involved in the study	
☐	☒	Antibodies
☐	☒	Eukaryotic cell lines
☒	☐	Palaeontology and archaeology
☐	☒	Animals and other organisms
☒	☐	Clinical data
☒	☐	Dual use research of concern
☒	☐	Plants

Methods

n/a	Involved in the study	
☒	☐	ChIP-seq
☐	☒	Flow cytometry
☒	☐	MRI-based neuroimaging

Antibodies

Antibodies used

For IHC:
p16 Abcam 211542
CD3 BIORAD MCA1477
CD8a Invitrogen 14-0808-82
GZMB Abcam ab4059
Ki67 Abcam ab16667
Pdpn R&D systems AF3244
CK8 PROGEN GP-K8
Vimentin PROGEN GP53
PDGFRA R&D systems AF1062
GFP abcam ab6673
DsRed (scarlet) Takara 632496
Bcl2 BD Biosciences 610539
α-SMA Sigma ab2547
BrdU BIORAD 1702
CK19 Abcam ab52625
BCL-W Cell Signalling 2724S
BCL-XL Cell Signalling 2764S
CD11b Abcam ab133357
Foxp3 Thermo Fisher Scientific 14-5773-82
Mcl-1 Proteintech 16225-1-AP
p16 Bio SB BSB 3479 1/50
CK18 Thermo MS-142-P 1/100
Vimentin Progen GP53 1/100

Large T-Ag BD-Pharmingen 554149
CD8a Invitrogen 14-0085-80 1/100
Mcm7 Santa Cruz sc-56324
CD45 abcam ab10558 1/150
CD45 BD-Pharmingen 550539 1/50
Mac-2 Biolegend 125401 1/50
cd31 R&D systems AF3628 1/100
Phospho-Stat1 Cell Signaling 9167S 1/100
RELA/NFκB p65 Santa Cruz sc-8008 1/500

For FACS:
Zombie Yellow BioLegend 77168
CD45 AF647 BioLegend 103123
CD45 PE BioLegend 103105
CD45 PECy5 BioLegend 103109
CD3e BUV395 BD Biosciences 563565
CD8a APC/Cy7 BioLegend 100713
CD11b PE/Cy7 BioLegend 101215
CD11c PB BioLegend 117321
MHC class II PE/Cy5 BioLegend 107611
Foxp3 PE BD Pharmingen 560414
Granzyme B PB BioLegend 515407
IFN-γ FITC BioLegend 505805
IFN-γ Pe/Cy7 BioLegend 505825
Ly-6C FITC BioLegend 128005
Ly-6G APC BioLegend 127613
F4/80 APC/Cy7 BioLegend 123117
PD1 PE/Cy5 BioLegend 135255
CD4 PE/Cy7 BioLegend 100527
CD4 FITC BioLegend 100527
EpCAM BV711 BioLegend 118233
CD31 PE BioLegend 102508
Pdpn BV421 BioLegend 127423
IFN-γ PE BD Biosciences 561056
CD8 BV711 BioLegend 344733
CD25 APC BioLegend 302609
CD4 APC/Cy7 BioLegend 317417
Granzyme B PB BioLegend 515407
CD90 PE/Cy7 BioLegend 328123
CD45 APC BioLegend 304011
EpCam BV711 BioLegend 324239
PDPN PE BioLegend 337003
PDGFRa (CD140a) PE/Cy5 BioLegend 135919 1/200

Validation

Anti-mouse p16 antibody was validated using p16 null mice. All other antibodies were validated based on manufacturer report, and locally using positive control tissues and cells.

Eukaryotic cell lines

Policy information about cell lines and Sex and Gender in Research

Cell line source(s)

Mouse CAFs were isolated from Kras-driven mouse PDACs. The PDAC primary carcinoma line X252 was established from a primary tumor grown in mice as a patient-derived xenograft, and was authenticated to the patient's short tandem repeat profile. Primary human CAF cultures were established from fresh digested PDAC tumors. Mouse KPC tumor cell lines 6422c1 and 6555c3 and F1199 were obtained from Ben Stanger and David Tuveson.

Authentication

Lines were not authenticated.

Mycoplasma contamination

All lines were tested routinely for mycoplasma and found negative.

Commonly misidentified lines
(See ICLAC register)

Name any commonly misidentified cell lines used in the study and provide a rationale for their use.

Animals and other research organisms

Policy information about studies involving animals; ARRIVE guidelines recommended for reporting animal research, and Sex and Gender in Research

Laboratory animals

Ptf1a-CreER (strain# 019378), C57Bl6), Kraslsl-G12D/+ (C57Bl6, strain# 008179), and p53flox/+ (strain# 008462, mixed background) were obtained from Jackson Laboratory and crossed to produce double and triple transgenic lines. The Ink-ATTAC transgenic line

	(C57Bl6) was obtained from Dr. Sheila Stewart with approval from Unity Biotechnology. Wild-type C57Bl6 mice were purchased from Jackson Laboratory (strain# 00664) and from Envigo. In transgenic Kras mice, experiments were initiated at 8 weeks of age, with tamoxifen injection to activate KRAS. Mice were sacrificed 4-10 months subsequently. In xenograft experiments mice were impanted with tumor cells at 6-8 weeks of age and sacrificed 3-4 weeks subsequently.
Wild animals	*Provide details on animals observed in or captured in the field; report species and age where possible. Describe how animals were caught and transported and what happened to captive animals after the study (if killed, explain why and describe method; if released, say where and when) OR state that the study did not involve wild animals.*
Reporting on sex	Both male and female mice were used in the experiments.
Field-collected samples	*For laboratory work with field-collected samples, describe all relevant parameters such as housing, maintenance, temperature, photoperiod and end-of-experiment protocol OR state that the study did not involve samples collected from the field.*
Ethics oversight	All experiments were conducted with approval from the Hebrew University Animal Care and Use Committee.

Note that full information on the approval of the study protocol must also be provided in the manuscript.

Flow Cytometry

Plots

Confirm that:

☒ The axis labels state the marker and fluorochrome used (e.g. CD4-FITC).

☒ The axis scales are clearly visible. Include numbers along axes only for bottom left plot of group (a 'group' is an analysis of identical markers).

☒ All plots are contour plots with outliers or pseudocolor plots.

☒ A numerical value for number of cells or percentage (with statistics) is provided.

Methodology

Sample preparation	For the preparation of single-cell suspensions, excised pancreatic tumors were minced with blades, incubated with Collagenase P (Sigma) and DNAse I (Sigma) for 30 min at 37°C, and filtered through a 100µM cell strainer. The cells were incubated with primary fluorophore-conjugated antibodies against surface markers for 30 minutes on ice in PBS containing 2% FBS. Intracellular staining was performed using the fixation/permeabilization transcription factor buffer set (BD Pharmingen) according to the manufacturer's instructions.
Instrument	Flow cytometric analysis was performed on an LSR Fortessa flow cytometer (BD Biosciences)
Software	Collection was done using BD FACSDiva, analysis was done using FCS Express.
Cell population abundance	Data appears within manuscript figures.
Gating strategy	Data appears within manuscript figures.

☒ Tick this box to confirm that a figure exemplifying the gating strategy is provided in the Supplementary Information.
